# Monitoring compartment-specific substrate cleavage by cathepsins B, K, L, and S at physiological pH and redox conditions

**DOI:** 10.1186/1471-2091-10-23

**Published:** 2009-09-22

**Authors:** Silvia Jordans, Saša Jenko-Kokalj, Nicole M Kühl, Sofia Tedelind, Wolfgang Sendt, Dieter Brömme, Dušan Turk, Klaudia Brix

**Affiliations:** 1 School of Engineering and Science, Jacobs University Bremen, Campus Ring 6, Research II, 28759 Bremen, Germany; 2 Department of Biochemistry and Molecular Biology, Jožef Stefan Institute, Jamova 39, 1000 Ljubljana, Slovenia; 3 Krankenhaus St. Joseph-Stift Bremen, Schwachhauser Heerstrasse 54, 28209 Bremen, Germany; 4 Department of Oral and Biological Sciences, Faculty of Dentistry, University of British Columbia, 2350 Health Sciences Mall, Vancouver, BC V6T 1Z3, Canada

## Abstract

**Background:**

Cysteine cathepsins are known to primarily cleave their substrates at reducing and acidic conditions within endo-lysosomes. Nevertheless, they have also been linked to extracellular proteolysis, that is, in oxidizing and neutral environments. Although the impact of reducing or oxidizing conditions on proteolytic activity is a key to understand physiological protease functions, redox conditions have only rarely been considered in routine enzyme activity assays. Therefore we developed an assay to test for proteolytic processing of a natural substrate by cysteine cathepsins which accounts for redox potentials and pH values corresponding to the conditions in the extracellular space in comparison to those within endo-lysosomes of mammalian cells.

**Results:**

The proteolytic potencies of cysteine cathepsins B, K, L and S towards thyroglobulin were analyzed under conditions simulating oxidizing versus reducing environments with neutral to acidic pH values. Thyroglobulin, the precursor molecule of thyroid hormones, was chosen as substrate, because it represents a natural target of cysteine cathepsins. Thyroglobulin processing involves thyroid hormone liberation which, under physiological circumstances, starts in the extracellular follicle lumen before being continued within endo-lysosomes. Our study shows that all cathepsins tested were capable of processing thyroglobulin at neutral and oxidizing conditions, although these are reportedly non-favorable for cysteine proteases. All analyzed cathepsins generated distinct fragments of thyroglobulin at extracellular versus endo-lysosomal conditions as demonstrated by SDS-PAGE followed by immunoblotting or N-terminal sequencing. Moreover, the thyroid hormone thyroxine was liberated by the action of cathepsin S at extracellular conditions, while cathepsins B, K and L worked most efficiently in this respect at endo-lysosomal conditions.

**Conclusion:**

The results revealed distinct cleavage patterns at all conditions analyzed, indicating compartment-specific processing of thyroglobulin by cysteine cathepsins. In particular, proteolytic activity of cathepsin S towards the substrate thyroglobulin can now be understood as instrumental for extracellular thyroid hormone liberation. Our study emphasizes that the proteolytic functions of cysteine cathepsins in the thyroid are not restricted to endo-lysosomes but include pivotal roles in extracellular substrate utilization. We conclude that understanding of the interplay and fine adjustment of protease networks *in vivo *is better approachable by simulating physiological conditions in protease activity assays.

## Background

Proteolytic processes are of vital importance due to the irreversibility of peptide bond cleavage. Spatio-temporal regulation of proteolysis is therefore of highest significance in determining the fate of cells. To date more than 66.500 gene sequences have been annotated as coding for proteolytic enzymes. The diversity of proteases is further reflected in broad tissue-, compartment- and substrate specificities. Most of all protease genes encode for serine peptidases (~25.000 sequences), followed by metallo (~23.000) and cysteine peptidases (~10.400) [1,2]. Because of this variety, one key issue of degradomics is to investigate networks of proteases in order to explain distinct proteolytic functions in the spatio-temporal context of substrate cleavage [3]. It is common practice to employ proteolytic activity assays to achieve this goal.

In this report we focused on cysteine cathepsins B, K, L and S, and analyzed their processing patterns using a natural substrate, thyroglobulin (Tg), while taking into account the suspected redox- and pH values of the physiological cleavage environments.

It has been shown before that the cysteine cathepsins B, K, L and S are localized to the endo-lysosomal system of thyroid epithelial cells. Moreover, they are present at extracellular locations such as the apical plasma membrane and within the follicular lumen of the thyroid gland [4-9]. Generally, cysteine cathepsins of the papain superfamily are well known to engage in bulk protein turnover at the increasingly acidic pH values of endocytic compartments [10]. Nevertheless, the proteolytic potency of cysteine cathepsins towards peptidic or natural substrates has been demonstrated to expand over a wide range including neutral pH values [4,11,12]. In fact, cysteine cathepsins were often accredited as extracellularly acting enzymes *in vivo *(for reviews, see [10,13]). Diverse protocols are available that describe methods to perform activity assays of cysteine cathepsins at neutral conditions, usually after activation of the proteases by incubation with reducing agents [14-19]. In reality, however, the extracellular space is not only a neutral but also an oxidizing environment, as much as endosomes and lysosomes exhibit not only acidic but also reducing conditions.

In this study, we have chosen redox-values of -220 mV to mimic the reducing environment of the cysteine-rich endosomes and lysosomes [20-22]. Moreover, endo-lysosomes are characterized by increasingly acidic conditions [23-25], and we considered pH 6.0 and pH 5.0 as closely reflecting the endosomal and lysosomal milieu, respectively. On the other hand, the thyroid follicle lumen represents a secluded compartment in which the tight junctions between thyroid epithelial cells tie up the extracellular storage space for Tg from the intercellular spaces between thyrocytes [26,27]. The follicle lumen is assumed to be neutral in pH thereby rendering Tg proteolysis by extracellular hydrolases unlikely (for reviews, see [26-28]). However, more recently we provided evidence for Tg proteolysis mediated by cysteine cathepsins within the extracellular thyroid follicle lumen before its endocytosis [6,7]. Extracellularly stored Tg of the human thyroid follicle lumen is covalently cross-linked by disulfide bonds [29] which can be formed in a self-assisted fashion via the CXXC-boxes within Tg [30], thereby attesting an oxidizing milieu within the thyroid follicle lumen. Furthermore, the direct pericellular environment adjacent to the apical plasma membrane of thyroid epithelial cells is oxidizing due to the presence of an H_2_O_2 _generating system which enables thyroid peroxidase to iodinate Tg at neutral pH [31,32]. We have chosen a value of -150 mV to mimic the extracellular space which is slightly more oxidizing than the range of -160 mV to -170 mV that was reported for the lumen of the endoplasmic reticulum [33].

**Figure 1:**
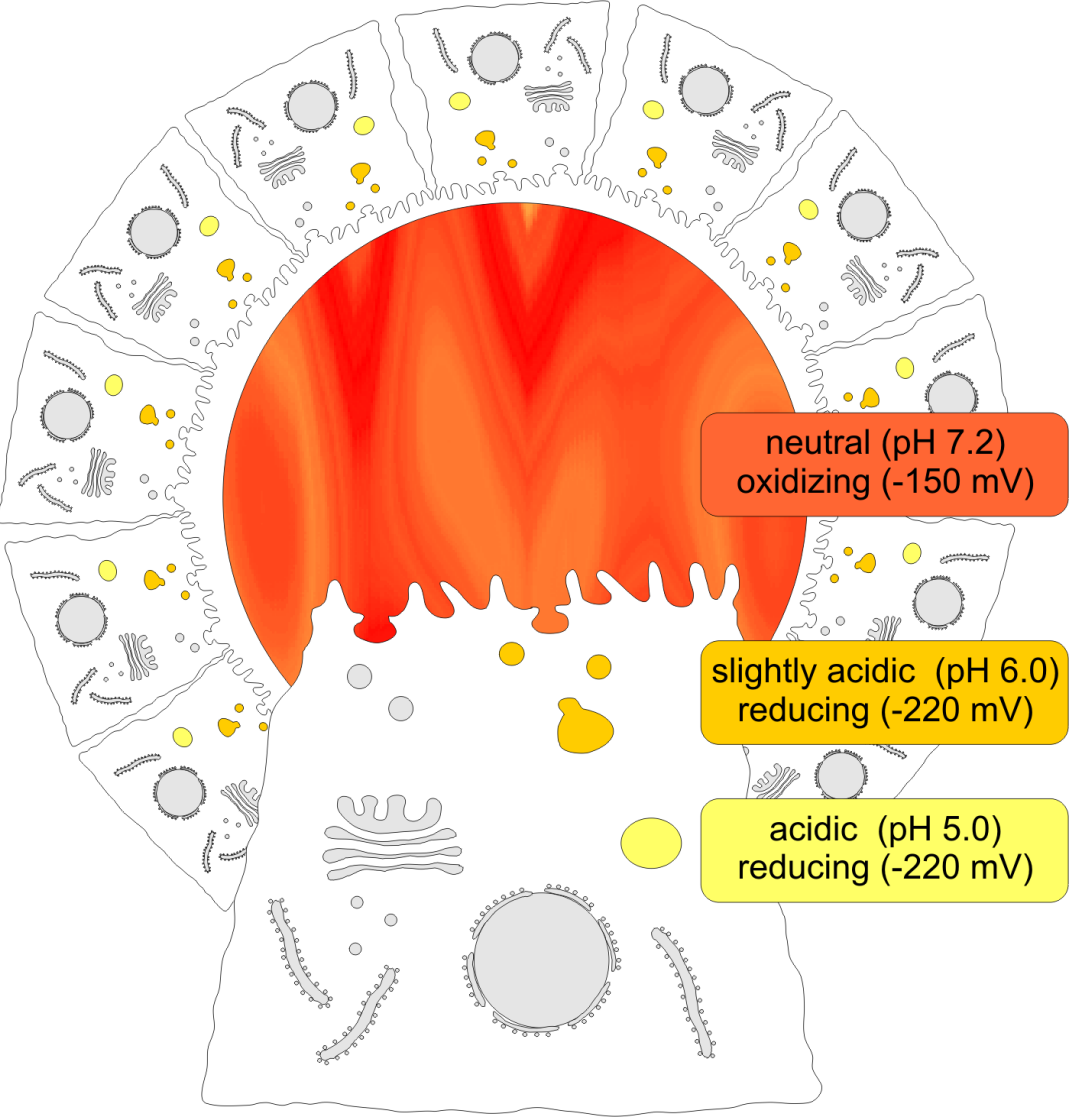
**Schematic overview representing the organization of a thyroid follicle**. The thyroid follicle lumen displays a protected extracellular compartment which is enclosed by a monolayer of thyroid epithelial cells interconnected by tight junctions. The apical plasma membrane of thyrocytes faces the follicle lumen in which compacted and covalently cross-linked thyroglobulin (Tg) is stored in globules reaching up to 150 μm in diameter. Tg-globules are far too large to be engulfed by single thyrocytes on the whole, thus partial degradation in the extracellular follicle lumen must precede endocytosis. By limited degradation, mediated by cysteine cathepsins within the pericellular space, Tg fragments are liberated and are subsequently endocytosed for degradation in endosomes and lysosomes. Thyroxine liberation by processing of the prohormone Tg starts extracellularly and is continued intracellularly after Tg-fragment internalization. Compartments in which proteolysis of Tg principally takes place are highlighted and the chosen redox- and pH-conditions were used as indicated in the associated boxes (for further details please refer to Background section).

As yet, there are only very few studies which consider both, pH values and redox potentials of the cleavage environment [21,34]. The assay described herein therefore employs buffers accounting for the conditions in the follicle lumen equivalent to the extracellular space or within endo-lysosomal compartments. We assessed the suitability of such buffers to promote proteolytic cleavage of Tg by specific cysteine cathepsins in order to simulate proteolysis within thyroid follicles, i.e. within an extracellular compartment, or within the endo-lysosomes of thyrocytes (Figure 1). Processing of Tg in the thyroid serves as an excellent model system for our purposes, because it has been demonstrated that (i) the cysteine cathepsins B, K, L and S cleave Tg *in vitro *[4,35-38], (ii) Tg is located in all compartments where cysteine cathepsins principally meet their substrates, i.e. endosomes, lysosomes, and in the extracellular space [[4,6-9], and this study], (iii) cysteine cathepsins and Tg represent a naturally occurring and physiologically relevant enzyme-substrate couple *in vivo *[6,7]. Under physiological conditions, proteolysis of Tg leading to thyroid hormone liberation is accomplished by cysteine cathepsins in a sequential process starting with the solubilization of Tg from its covalently cross-linked macromolecular storage forms that fill the extracellular follicle lumen. The initial substrate cleavage results in the production of smaller protein fragments that are internalized by thyroid epithelial cells together with partially processed Tg [6,7]. Accordingly, liberation of the thyroid hormones T_3 _and T_4_, which are essential for the regulation of mammalian development and metabolism begins extracellularly and is perpetuated within endocytic compartments of thyroid epithelial cells.

This study aims at elucidating crucial roles of selected cysteine cathepsins in this sequence of prohormone processing. The development of an *in vitro *assay system mimicking the *in situ *pH and redox conditions enabled analysis of the potency of cathepsins B, K, L and S to cleave their natural substrate Tg and to liberate thyroid hormones under conditions simulating favorable (endo-lysosomal) versus non-favorable (extracellular) compartments.

## Results

### Localization of cysteine cathepsins in the human thyroid gland

**Figure 2:**
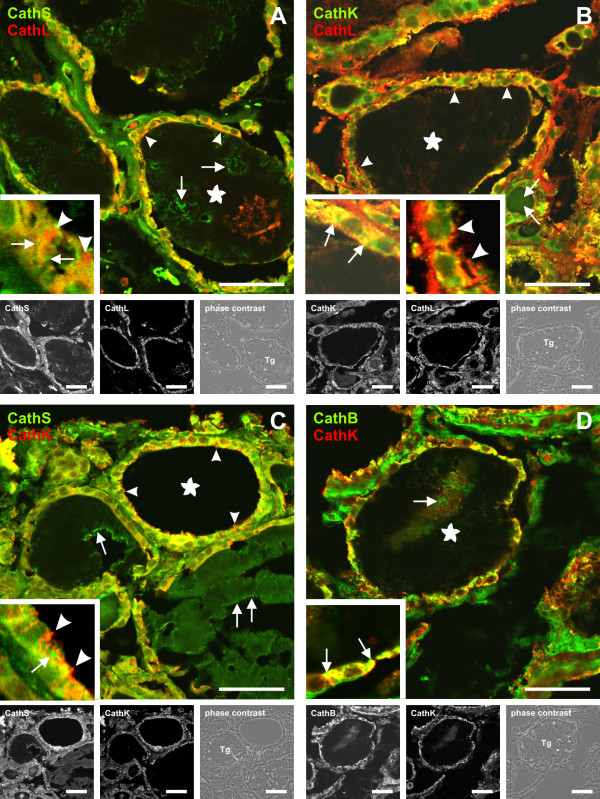
**Localization of cysteine cathepsins in human thyroid tissue**. Multi-channel fluorescence (A-D and insets), single channel and corresponding phase contrast micrographs (black and white) of cryo-sections taken from human thyroid tissue that were immunolabeled with antibodies directed against cysteine cathepsins B, K, L, or S in different combinations, as indicated. Co-localization is displayed by yellow signals as a result of overlapping red and green signals for either antibody labeling. Cathepsins B and K as well as K and L were mainly co-localized to the same intracellular compartments (B and D, insets, arrows), whereas cathepsin S was found in structures that were lacking other cysteine cathepsins (A and C, insets, arrows). Likewise, vesicles containing only cathepsin L or K (A and C, insets, arrowheads) were also prominent. All cysteine cathepsins were present within the Tg-containing extracellular follicle lumen (asterisks, Tg) where no co-localization of either cysteine cathepsin with one another was detectable. Cathepsins K and L were further detected in association with the apical plasma membrane of thyrocytes (B and C, insets, arrowheads). Bars represent 50 μm.

The subcellular localization of cysteine cathepsins has been studied in detail in almost all tissues but not to the same extent in the human thyroid gland. Therefore and in order to analyze the distribution patterns of particular cysteine cathepsins in the compartments of Tg processing, human thyroid tissue was immunolabeled with antibodies against cathepsins B, K, L, and S in single and double labeling experiments. Cathepsins B, K, and L were co-localized within the same intracellular vesicles (Figure 2B and 2D, yellow signals). Cathepsin S appeared to be also localized to distinct vesicular compartments, because co-localization with cathepsins K and L was not as prominent (Figure 2A and 2C). Some cathepsin L-positive vesicles were even lacking cathepsin S (Figure 2A, inset, arrowheads). Likewise, cathepsin K-positive structures were often negative for cathepsin S (Figure 2C, inset, arrowheads). Consequently, cathepsin S-positive vesicles lacking cathepsins K or L were frequently detected (Figure 2A and 2C, insets, arrows). Moreover, all cathepsins analyzed were detected in the extracellular space, i.e. within the follicle lumen, indicating their secretion (Figure 2, arrows in lumina labeled with asterisks). Note, that the extracellular follicle lumen (Figure 2, asterisks) represents a storage compartment for Tg, hence, all cathepsins are found in close proximity to their natural substrate already here. Cathepsin S was prominently present in the extracellular follicle lumen (Figure 2A and 2C, arrows in lumina labeled with asterisks). Cathepsins K and L were additionally found pericellularly in close association with the apical plasma membrane (Figure 2B and 2C, arrowheads), where thyroid hormone liberation by Tg processing is believed to start. We conclude that trafficking of proteases in thyroid epithelial cells results in a distinct protease composition within vesicles of the endocytic pathway in addition to their secretion into the extracellular space. Hence, it is important to analyze Tg processing with different combinations of cysteine cathepsins and to simulate substrate cleavage at the conditions of the respective compartments, i.e. extracellular follicle space, endosomes and lysosomes.

### Establishment of buffers with stable redox potentials at distinct pH values

**Figure 3:**
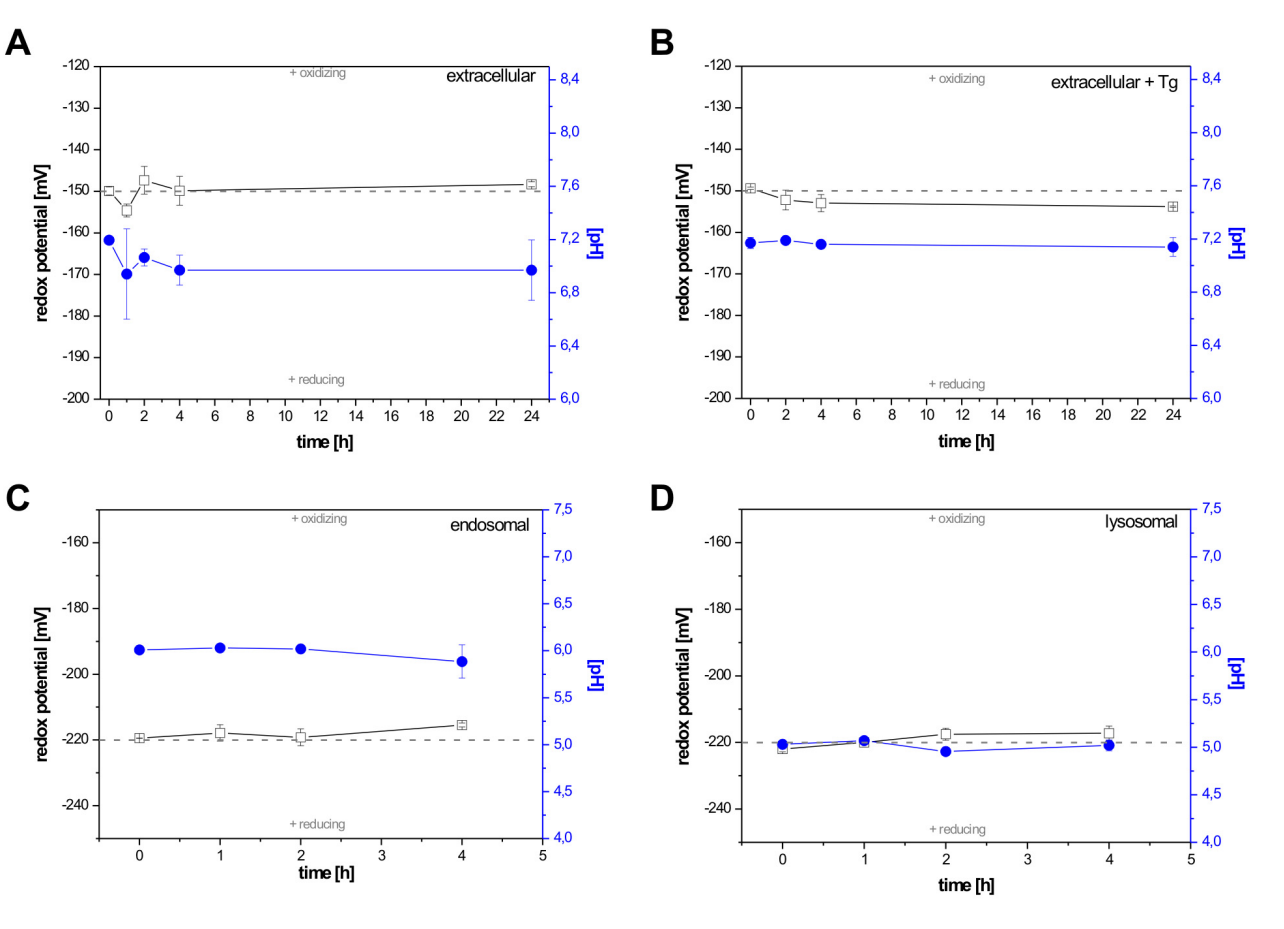
**Stability of assay buffers**. Redox potentials [mV] (open squares) and pH values (filled circles) were measured for the indicated time intervals. A two-buffer system of citric acid and sodium phosphate was used to adjust the desired pH values while the redox values were adjusted by the addition of L-cysteine. Timepoint [t = 0] reflects the initially adjusted value; the setpoint of the redox potential is given as dashed line. Redox values above the setpoint are considered more oxidizing, whereas changes towards the negative potential are more reducing. Buffers representative for the extracellular space, i.e. mimicking an oxidizing (-150 mV) and neutral (pH 7.2) compartment, were measured in the absence (A) or presence (B) of the substrate Tg. Buffers mimicking the endosomal compartment were characterized by reducing (-220 mV) and slightly acidic (pH 6.0) conditions (C). Buffers representing the lysosomes displayed the same reducing redox-conditions as endosomes (c.f. C), but were more acidic (pH 5.0) (D). Values are given as mean +/- SD.

Buffers representative for the extracellular space, the endosomal compartment and for lysosomes were established through the use of a two-buffer system of citric acid, sodium phosphate, and L-cysteine. In order to verify buffer stability, the actual pH and redox values were determined for a minimum time period of 24 hours. The buffer simulating extracellular conditions with an oxidizing redox potential of -150 mV and neutral pH 7.2 was shown to be stable over 24 hours (Figure 3A). Although addition of Tg marginally lowered redox and pH values, the overall stability under extracellular conditions was more stringent in the presence of substrate (Figure 3B). Buffers mimicking endosomes and lysosomes were adjusted to -220 mV and differed from one another in their pH values, only. The endosomal-representative buffer was set to pH 6.0 and the lysosomal to pH 5.0. The stability of these buffers with respect to redox potential and pH value was verified over a 4 h time period (Figure 3C and 3D) as these buffers were stable from the very beginning.

### Determination of cleavage sites within Tg

**Figure 4:**
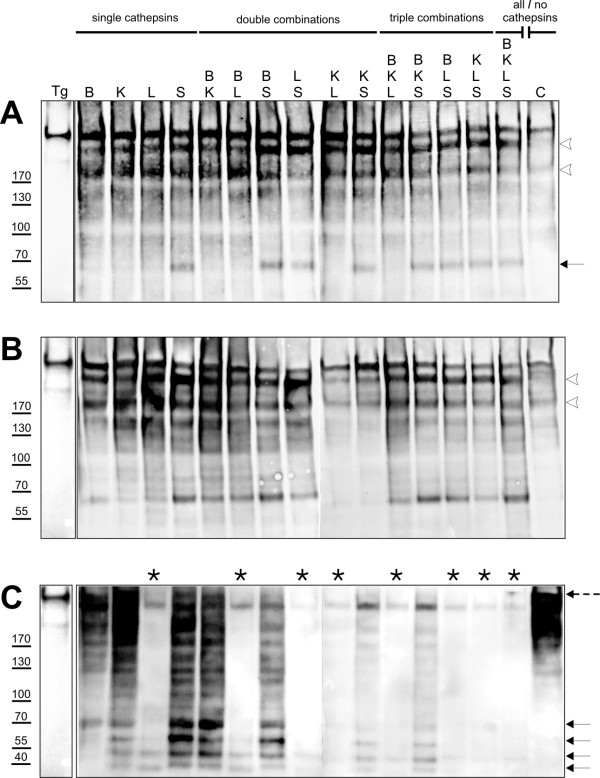
**Cleavage efficiency of cysteine cathepsins at distinct subcellular conditions**. Single cathepsins or combinations thereof were incubated with thyroglobulin (Tg) *in vitro *in buffers representative of extracellular conditions (A), endosomes (B) or lysosomes (C) for 2 hours at 40°C. As a control, purified Tg was incubated in the respective buffers without proteases (A-C, last lanes). Samples were PAGE-separated and Tg fragments were visualized by immunoblotting. Efficiency of cleavage at neutral and oxidizing conditions was indicated by the appearance of a 250 kDa and a 200 kDa fragment (A, arrowheads). Similar fragments were observed under endosome-representative conditions (B, arrowheads). Most effective substrate degradation, discernible by numerous low molecular weight fragments, was observed under reducing, acidic conditions mimicking lysosomes (C). Cathepsin L can be considered most effective in Tg-processing within the lysosomal compartment, because samples containing this protease (C, asterisks) featured numerous low molecular weight fragments (arrows) and almost lacked intact, full-length Tg (dashed arrow). Note, that under neutral and oxidizing conditions, cathepsin S qualified to be most effective in Tg-processing. All samples including this protease featured a specific 70 kDa fragment (A, arrow) additionally to higher molecular mass products. In contrast to extracellular or lysosomal conditions, all peptidases tested performed equally well at endosome-representative conditions.

**Figure 5:**
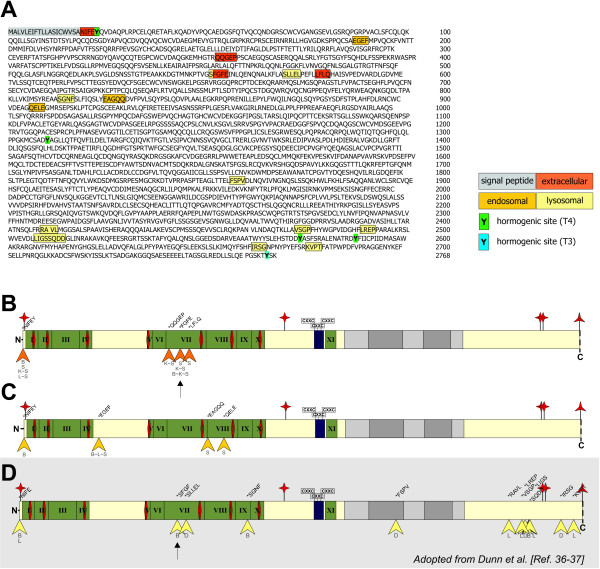
**Preferential cleavage sites for cysteine cathepsins within the thyroglobulin sequence at distinct redox- and pH-conditions**. Characteristic Tg-fragments obtained by *in vitro *degradation at extracellular or intra-endo-lysosomal conditions were N-terminally sequenced. Newly generated N-termini are highlighted (A); box colors represent the respective condition at which fragments were obtained (c.f. Figure 1). Hormogenic sites, i.e. pre-formed thyroid hormones tri-iodothyronine (T_3_) and thyroxine (T_4_), are tagged in blue and green; the signal peptide is highlighted in grey. (B-D) Schematic drawings sketch the Tg molecule that comprises Tg Type I domains (green), Type II (blue), Type III A (light grey), Type III B repeats (dark grey), Thyropins (red), and CXXC-motifs (see [6] for details). Arrowheads indicate relative positions of identified cleavage sites utilized in extracellular- (B) or endosomal-like Tg processing (C). Cleavage sites of cathepsins B, D, and L were determined by N-terminal sequencing of Tg fragments derived from incubation of rabbit Tg with lysosomal extracts or cathepsins isolated from human thyrocytes (D; according to [36,37]). This scheme (D) is shown for comparison, only, and shaded to indicate that it is adopted (see [6]) from the results of studies performed by Dunn and co-workers [36,37]. Corresponding cysteine cathepsins or combinations thereof are given below the arrowheads; the respective neo-N-termini are specified above the representative schematic drawing. Arrows in B and D indicate similar cleavage sites under extracellular conditions and within lysosomes, respectively.

Isolated Tg was incubated with cathepsins in buffers simulating extracellular conditions and the extent of substrate cleavage was compared to that achieved under conditions characteristic for intracellular proteolysis within endo-lysosomal compartments. Cathepsins B, K, L and S or combinations thereof were incubated with Tg in the respective buffers and proteolysis was allowed to occur for 2 hours. Preparations were then heated in LDS sample buffer without any further addition of reducing agents. This procedure ensured consistent behavior of all samples from the same conditions upon separation by PAGE since they were run on separate gels. Fragmentation patterns were analyzed by SDS-PAGE followed by immunoblotting using antibodies specific for Tg (Figure 4) and by N-terminal sequencing of selected Tg-fragments (Figure 5). Note, that for all incubations an identical amount of Tg was subjected to cysteine cathepsin degradation, whereby the particular amount of distinct enzymes was kept constant in all incubations. Therefore, the ratios of total enzyme to substrate differed between single and combinatorial incubations (see also Materials and Methods). We were especially decided about such conditions, because we consider this setup to best reflect Tg cleavage *in situ*. As we have shown in the localization studies (see Figure 1), vesicles may contain only one type of enzyme or combinations of different enzymes. For the extracellular follicle lumen, similar approximations might be relevant, since we observed almost no co-localization of the distinct cysteine cathepsins in the extracellular space, but different enzymes were detectable and can therefore, in principle, cleave luminal Tg. Hence, the observed degradation patterns must be interpreted as resulting from different combinations of enzymes acting sequentially and/or at the same time on Tg.

Under neutral and oxidizing conditions mimicking the extracellular thyroid follicle lumen full-length Tg of approximately 330 kDa was cleaved by each of the cysteine cathepsins tested, even though these buffer conditions are considered as 'non-favorable' for proteolytic action of cysteine cathepsins (Figure 4A). The effectiveness of substrate-cleavage was obvious from the appearance of two fragments of approximately 250 and 200 kDa (Figure 4A, arrowheads), which were also detected when the assay buffers reflected more favorable endosomal cleavage conditions, i.e. a reducing and slightly acidic environment (Figure 4B, arrowheads). In addition to the high molecular mass fragments, all samples containing cathepsin S displayed a specific 70 kDa fragment under extracellular conditions (Figure 4A, arrow). The same-sized fragment was generated at conditions characteristic for endosomes and lysosomes by several combinations of cysteine cathepsins but notably not by cathepsin K alone or in combination with cathepsins L and S. The analysis of degradation patterns using assay conditions mimicking lysosomal cleavage revealed a number of low molecular mass fragments of Tg as generated by all cathepsin combinations (Figure 4C, arrows). Samples containing cathepsin L lacked the intact, full-length Tg (Figure 4C, dashed arrow), but contained numerous smaller fragments of 40-70 kDa (Figure 4C, asterisks). Note that all preparations contained equal amounts of Tg at the start of incubation with cysteine cathepsins under different conditions. The lanes were loaded after the incubation period with identically sized aliquots from each preparation. Hence, the absence of signal in certain lanes of the immunoblots is representative of extensive Tg degradation rather than being the result of unequal loading of the gels. Accordingly, blank or weakly labeled lanes indicate complete degradation of Tg by the respective cysteine cathepsin/s at these conditions. As a control of its stability, Tg was incubated in the respective buffers, but without any protease added (Figure 4A-C, last lanes).

In order to determine the cleavage sites that were used by cysteine cathepsins, characteristic Tg fragments were N-terminally sequenced (Figure 5). A band with a molecular mass of approximately 330 kDa exhibited the same N-terminus as unprocessed Tg in all instances, thus representing intact, full-length protein. Tg-fragments originating from proteolytic processing by cathepsin S under extracellular conditions had molecular masses of approximately 200 kDa, 170 kDa and 70 kDa. Two of these fragments exhibited newly generated N-termini, while the 70 kDa fragment showed the same N-terminus as unprocessed Tg. Arrowheads indicate relative positions of identified cleavage sites as deduced from N-terminal sequencing of extracellular- (Figure 5B) or endosomal-like Tg degradation (Figure 5C). In Figure 5D a scheme of lysosomal cleavage sites is depicted, which was determined by Dunn and colleagues [36,37] under near-physiological conditions since lysosomal extracts were used to degrade Tg. The schematic drawings in Figure 5 illustrate that the cleavage sites varied within Tg depending on the conditions under which cleavage took place.

### Analysis of physiological significance of Tg cleavage

**Figure 6:**
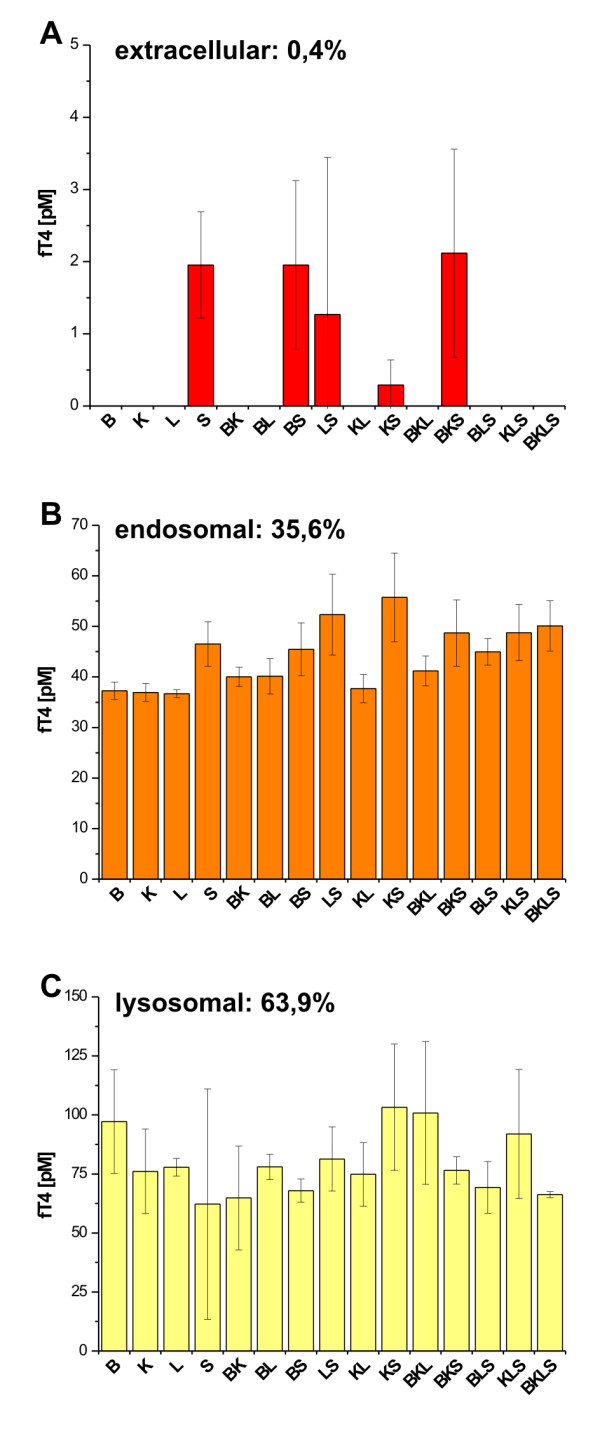
**Cysteine cathepsins efficiency to liberate thyroxine (T_4_) *in vitro***. Samples taken after 2h-*in-vitro*-degradation of Tg were assayed for their content in free thyroxine (fT_4_) by ELISA. Efficiency of hormone liberation by distinct cysteine cathepsins or combinations thereof is directly correlated to the amount of fT_4_. Under conditions mimicking the extracellular space, exclusively cathepsin S proved to be efficient in thyroxine liberation (A), whereas under endosomal-representative conditions all cysteine cathepsins performed equally well (B). The same holds true for conditions mimicking the lysosomal compartment, although cathepsin S was less active as reflected by lower thyroxine liberation potency as compared with e.g. cathepsin B (C). Highest amounts of fT_4 _were measured for lysosomal-mimicking environments, followed by those performed under conditions simulating endosomes and the extracellular space (A-C). Values are given as mean values +/- SD.

Determination of cathepsin cleavage sites revealed that the N-terminal half of Tg was prone to extracellular and endosomal cleavage, whereas the thyroid hormone-rich C-terminal half is, according to [36,37], best cleaved under conditions of lysosomal Tg processing. In order to implement a physiologically relevant read-out for efficiency of substrate cleavage, we analyzed the *in vitro*-degradation samples for their content of liberated, free thyroxine (fT_4_) by ELISA. Under extracellular conditions, fT_4 _was only detected in samples that contained cathepsin S alone or in combinations with other cathepsins (Figure 6A). Under endosomal conditions, the overall efficiency of fT_4 _liberation was considerable, about 70 fold higher than compared to extracellular conditions with values of fT_4 _ranging from approximately 35 to 55 pM (Figure 6B). Yet, another augmentation in the amount of liberated T_4 _was observed under conditions simulating lysosomes where cathepsins B, K and L performed equally well by liberating T_4 _in a range from 75 to 100 pM (Figure 6C).

## Discussion

One important mechanism to control protease activity is given by endogenous inhibitors, another is represented by the dependency of proteolytic enzymes on the pH and redox potential of the cleavage environment. Whereas pH sensitivity has been studied quite extensively (for review, see [39]), much less is known about their sensitivity to the redox potential of the environment. Endo-lysosomal cysteine proteases like cathepsins L and B are considered highly instable at neutral pH values with half lives of 1.3 and 15 minutes, respectively [40,41]. The inactivation appears to be irreversible and is associated with a loss of native 3D structure. However, a few other cysteine cathepsins of the papain superfamily clan C1A display prolonged stability and activity at neutral pH values, cathepsins K and S are the most prominent examples [4,11,18]. In contrast, papain as well as cruzain exhibit stability at broad ranges of pH values and withstand neutral to even basic pH-conditions [42,43]. On the other hand, other plant proteases are particularly prone to turn instable at pH values differing from their acidic optimum [43]. These pronounced differences in the pH-dependent stability indicate that papain-like enzymes have evolved [44,45] to play roles in a variety of biological processes, bringing proteolysis into milieus outside acidic endo-lysosomal compartments. Therefore, mammalian cysteine cathepsins might be the "specialists" in acidic environments, although they are still suited to perform their actions, at least temporarily, under the neutral pH-conditions of the extracellular space that are nowadays considered non-favorable or even "extreme" [5,10,46].

In fact, proteolytic activity of lysosomal cysteine peptidases in an environment exhibiting conditions opposite to the reducing and acidic milieu of endo-lysosomal compartments was not long ago considered to be a mission impossible. Oxidizing conditions enforce the formation of disulfide bonds by reactive thiol groups which makes the active site cysteine of cathepsins highly sensitive to oxidation. Thus, under reducing conditions, like it is assumed for the endo-lysosomal compartment, the activity of cysteine cathepsins should be considerably higher than in other compartments exhibiting oxidizing conditions, like e.g. the extracellular space. In the thyroid, however, the extracellular follicle lumen is the scene of action for the initial steps of thyroid hormone utilization, i.e. the solubilization of covalently cross-linked Tg and thyroxine liberation by proteolytic processing mediated primarily through cysteine cathepsins B, K, L and S [7].

Up to now, the hypothesis that cysteine peptidases hold proteolytic activity in oxidizing environments was rarely challenged experimentally due to the lack of suitable activity assays with conditions mimicking the *in vivo *situation in the most life-like fashion, i.e. at realistic pH and redox conditions in physiological settings. *In situ*, redox conditions in cellular compartments or in the extracellular space are achieved and maintained by redox pairs of which cysteine(Cys)-cystine(CySS) constitutes the prominent and natural redox-pair of the extracellular space and endo-lysosomal compartments [47]. In keeping with this notion, exact values describing redox potentials are astonishingly scarce and have been reported for the cytosol (-221 mV to -236 mV) and the ER lumen (-160 mV to -170 mV) [21,22,33]. The latter represents an oxidizing intracellular compartment of protein folding in which disulfide bridges are formed and maintained, whereas the cytosol is clearly reducing. For the choice of our buffer system we used the (Cys)-(CySS) redox-pair to adjust the redox potential and we considered a redox potential of -220 mV suitable to simulate reducing conditions of endosomes and lysosomes. A redox value of -150 mV was adjusted for mimicking the extracellular space, i.e. we assumed a slightly more oxidizing milieu for the extracellular space than that observed for the ER lumen. Through the use of a two-buffer system with citric acid and sodium phosphate and through the addition of L-cysteine, we have established buffers with stable redox potentials at distinct pH values for *in vitro *analyses of proteolytic substrate processing potency.

Another critical factor in proteolytic profiling is the choice of substrate. Commonly, small synthetic substrates are used for standard protease activity assays. More recently it became mandatory to use natural substrates in *in vitro *assays to collect more appropriate data; especially considering that protease-substrate interaction may also affect enzyme activity [3]. The cleavage of Tg by cathepsins is a naturally occurring process of high physiological importance [7], which makes Tg an excellent substrate in our proteolytic assays. The partial degradation of extracellularly stored full-length Tg within the thyroid follicle lumen is of great importance *in vivo*. It represents the necessary first step to produce smaller fragments of the pro-hormone that can easily be endocytosed to ensure endo-lysosomal processing and efficient thyroid hormone liberation. In the *in vitro *assay presented herein, partial proteolytic processing of full length Tg was achieved by all cathepsins, resulting in the generation of three distinct fragments even at neutral and oxidizing conditions. The abundance of low molecular mass Tg-fragments in lysosomal-mimicking conditions of this assay must be considered representative of an extensive overall cleavage of the substrate. It can therefore be deduced that all cysteine cathepsins tested exhibited highest proteolytic activities, indeed, under acidic and reducing conditions. This notion is in line with the catabolic function of cysteine cathepsins *in situ *which is mainly conducted within the endo-lysosomal compartment. We consider cathepsin L as the most potent peptidase to catabolize Tg within the lysosomes, because the respective samples lacked full-length, intact Tg almost entirely, but contained numerous smaller fragments of 40-70 kDa.

The application of several cathepsins at once proved to be efficient in well-directed substrate cleavage further strengthening results from our previous *in vivo *studies in which it was shown that a concerted action of cathepsins K and L is needed for T_4 _liberation *in vivo *[7]. The results presented herein further support the conclusion that Tg-processing and thyroid hormone liberation is achieved by proteases acting consecutively towards efficient pro-hormone utilization. Cathepsin S proved to be the best-suited cysteine cathepsin to extracellularly liberate T_4 _from Tg, i.e. to conduct hormone liberation before the pro-hormone eventually reached endosomes and lysosomes. Cathepsin S was also able to liberate T_4 _under endosomal and lysosomal conditions, but then, it was not any more efficient than the other cysteine cathepsins tested. In fact, it has been shown that cathepsin S is the only cysteine cathepsin which is truly stable at neutral pH when analyzed under reducing conditions, whereas cathepsins K and L are unstable and cathepsin B exhibits limited stability at reducing and neutral pH conditions [11,17,18,40,41,48]. Interestingly, an additional cleavage site for the combination of cathepsins K and S was determined within the N-terminal half of Tg at extracellular conditions as compared to cleavage by cathepsin S alone (Figure 5B). This might explain why liberation of T_4 _under these conditions was less effective when both, cathepsins K and S were used in contrast to combinations of cathepsin S with cathepsins B or L, in which the cathepsin S ability to utilize T_4 _from Tg clearly dominated (Figure 6A). However, it should be kept in mind that the assay conditions as presented herein differ significantly from those of previous studies in that we mimicked physiological conditions by testing cysteine cathepsins under oxidizing and neutral pH conditions. Therefore, our results gained by studying cleavage of their natural substrate Tg under such comparatively "extreme" non-favorable conditions clearly differ from the standard assay conditions in which the read-out is not physiological, but instead cleavage of synthetic substrates is monitored. Hence, we believe that the data presented here has to be interpreted on the background of physiological protein processing with the biologically significant read-out, that is, utilization of T_4 _by processing of the precursor Tg.

None of the cleavage sites used by any of the cysteine cathepsins tested was identical in both, extracellular and endosomal conditions, i.e. in the compartments of well-directed substrate processing. Thus, we conclude that preferential cleavage is largely up to the compartment in which substrate processing takes place. The presence of different sets of proteolytic enzymes in such compartments may allow for a combinatorial and sequential substrate cleavage. This is to say that specific cleavage sites are used in distinct environments, which provide stable, biochemically defined conditions for specific sets of enzymes, but do not necessarily exhibit the most favorable conditions for bulk proteolytic processing. This notion is further supported by comparative analysis of endosomal-typical cleavage with respect of lysosomal-typical cleavage sites, which completely lack analogies as has been determined by us (this study) and by Dunn and co-workers using isolated cathepsins or extracts of thyroid lysosomes [36,37]. Even though the cysteine cathepsins B and L were included in both studies, only one cleavage-site (Figure 5B and 5D, arrows) was found to be alike in extracellular conditions (this study) and in Tg fragments identified in lysosomal extracts (studies by Dunn and colleagues [36,37]). Interestingly, this particular cleavage site was not reproduced by identical cathepsins in different environments, but the extracellular-representative cleavage of Tg by cathepsin S (this study) generated an almost identical neo-N-terminus as the cathepsin B-mediated cleavage determined by Dunn and co-workers [36,37]. Thus, although the amino acid sequence of the substrate itself predominantly dictates its cleavage, the specificity of substrate cleavage is additionally directed by the biochemical environment of cleavage and by the set of actually cleaving enzymes.

It is important to keep in mind that the extracellularly acting cysteine cathepsins in the thyroid follicle are derived from their secretion into the follicle lumen after being recruited out of endo-lysosomal compartments [9]. It is generally accepted that the proteolytic activity of such already activated cysteine peptidases can be maintained under oxidizing conditions in the extracellular space for longer time intervals [10,34,49]. Since the proteolytic enzymes cleaving Tg in the thyroid follicle lumen have experienced a reducing and acidic environment for proper processing and activation before being secreted, they may as well contribute significantly to physiological thyroid hormone liberation by extracellular proteolysis of Tg.

## Conclusion

Our study shows that a specific subcellular location is the key figure in the nature of proteolytic processing, i.e. timing and location of peptidase activities determine whether bulk protein turnover or, instead, specific substrate processing takes place. Importantly enough, the onset of many pathological processes like tumor cell metastasis, rheumatoid arthritis, pancreatitis or multiple sclerosis goes along with alterations in the patterns of cysteine cathepsin localization and with pericellular acidification which may well be suited to prolong the extracellular actions of these proteases (for reviews see [10,13,50]).

The routine to analyze cysteine cathepsin-mediated processing of Tg presented herein will be easily adaptable to investigate any protease's proteolytic potential towards any natural or artificial substrate. Hence, our approach is applicable in order to study the degradome in a physiological context at simulated *in vivo *conditions. By adapting our assay system to other proteases, we anticipate a better understanding of the interplay and fine-adjustments of protease-networks that control substrate processing in the determination of 'vital' or 'fatal' changes in cellular destiny. Comprehensive knowledge of proteolysis in physiological or pathological conditions, in turn, is critical for the development of effective treatments and novel therapeutics.

## Methods

### Depletion of non-covalently associated T4 from isolated human Tg

Purified human Tg [29,51] was mixed with 200 μg/mL 8-anillino-1-naphtalene-sulfonic acid ammonium salt (ANS, Calbiochem/Merck, Darmstadt, Germany) and incubated on an end-over-end-rotator (14 rpm) for 1 h in order to dissociate non-covalently bound T_4 _from its precursor that is known to eventually function as a T_4_-binding protein. The mixture was cleared of free T_4 _by two subsequent incubations with an anion exchange resin, DOWEX 1 (1 × 8 chloride form, 200-400 mesh; 50 mg/mL; Sigma, Munich, Germany). The crude and fine resin parts were removed by two centrifugation steps at 1,000 and 30,000 × g for 10 and 20 minutes, respectively. Supernatants were used for protein determination according to Neuhoff [52].

### Preparation of assay buffers

A two-component buffer system of (A) 200 mM citric acid and (B) 200 mM dibasic sodium phosphate was used. Specific volumes of (A) and (B) were mixed as indicated below and buffers were pre-warmed to 40°C (according to [14]) while constantly stirring. Redox potentials were adjusted by the addition of L-cysteine (Roth, Karlsruhe, Germany) in the indicated amounts. Adequacy and stability of pH and redox values were constantly monitored using a pH electrode (SenTix 81) and an electrode for redox control (SenTix ORP), both connected to an InoLab pH Level 1 (all WTW, Weilheim, Germany). The pH of 7.2 in the buffer simulating the extracellular space was achieved by mixing 6.9 mL of buffer A and 87.1 mL of buffer B. Upon the addition of 9.58 mg L-cysteine the redox potential of the buffer shifted to -150 mV. The buffers mimicking slightly acidic endosomal compartments (pH 6.0, -220 mV) and acidic lysosomal compartments (pH 5.0, -220 mV) were prepared by mixing 19.7 mL of buffer A and 68.3 mL of buffer B with 190 mg L-cysteine, or 28 mL of buffer A with 65.5 mL of buffer B and 2.2 g L-cysteine, respectively. Buffers were equilibrated at assay temperature before use.

### *In vitro *Tg degradation assay

Cathepsins B and L, purified from human liver, were purchased from Calbiochem (Darmstadt, Germany), whereas human cathepsins K and S were recombinant enzymes [11,18]. Human Tg and single cathepsins or combinations thereof were mixed in 100 μL of buffers mimicking extracellular, endosomal or lysosomal conditions to a final concentration of 9.5 pM for Tg, and 4.75 pM of either of the cathepsins. Therefore, the substrate to peptidase ratios varied from 1:0.5 up to 1:2 in single, double, triple, and quadruple cathepsin combinations. Samples were prepared in LowBind protein tubes (Eppendorf, Hamburg, Germany), spun down and incubated on an end-over-end rotator (40 rpm) for 2 hours at 40°C in a heated incubator. Then, samples were briefly centrifuged and processing of Tg was stopped either by freezing 50 μL aliquots of the samples at -20°C or by direct preparation of the samples for subsequent SDS-PAGE analysis. Samples were mixed with lithium dodecyl sulfate (LDS) sample buffer, heated to 70°C for 10 minutes, and 50 μL of each individual sample was loaded per lane. Gel-electrophoresis was performed in an XCell SureLock Mini-Cell using pre-cast 3-8% NuPAGE Novex Tris-Acetate gels and NuPAGE Tris-Acetate SDS running buffer (all Invitrogen, Karlsruhe, Germany) at 150 V for 75 minutes at RT. To ensure consistent running behavior, gels were loaded with samples from either of the three conditions so that reducing and non-reducing samples would not affect each other during separation. Subsequently, Tg and fragments were blotted onto polyvinylidenfluoride (PVDF) membranes at 30 V for 60 minutes using the XCell II Blotting Module and NuPAGE transfer buffer (all Invitrogen) according to the manufacturer's instructions.

### Immunodetection of Tg and its degradation fragments

Membranes were blocked using 5% non-fat milk powder in PBS-Tween (0.34 M NaCl, 0.316 M Na_2_HPO_4_, 58.49 NaH_2_PO_4_, pH 7.2, supplemented with 0.3% Tween-20). Primary antibodies directed against bovine Tg [51] were diluted 1:1,000 in 2.5% non-fat milk powder and membranes were incubated 90 minutes at RT. Secondary, horseradish peroxidase (HRP) -coupled antibodies were allowed to bind for 60 minutes at RT. Immunoreactions were visualized by enhanced chemiluminescence (ECL) and imaged on CL-XPosure Clear Blue X-ray films (both Pierce, PerbioScience, Bonn, Germany) which were scanned using a transmitted light scanner device (OpticPro ST48, Plustek, Norderstedt, Germany).

### N-terminal sequencing of separated Tg fragments

For sequencing, the degradation assays, gel-electrophoresis, and blotting were performed as above. The absolute content of cathepsins and Tg, however, was increased four-fold. PVDF membranes were briefly washed in ddH_2_O and stained overnight with 0.1% Coomassie R250 in 1% acetic acid and 40% methanol. Background was removed by washing in 50% methanol until bands became clearly visible. Selected protein bands showing adequate staining were cut and stored at -20°C until further analyzed by automated sequence analyses on a Procise pulsed-liquid protein sequencer (Applied Biosystems, Foster City, CA, USA).

### Determination of thyroid hormone liberation by fT4-ELISA

To assess the amount of liberated thyroxine, 50 μL of the *in vitro *degradation samples were analyzed using a commercially available solid phase-based competitive immunoassay (DRG Diagnostics, Marburg, Germany). Serum samples that were included in the ELISA kit and contained defined fT_4_-amounts were used as standards. Serum standards and *in vitro *degradation samples were assayed in duplicates and treated equally in all respects. The assay was conducted according to the manufacturer's recommendation with the performance of optional washes (10 × 5 minutes) with ddH_2_O. Immediately after addition of the color reactant the absorbance at 450 nm was read in a microplate reader (MRX Microplate Reader, Dynatech Laboratories Inc., Chantilly, VA, USA). The fT_4 _amount calculated for blank values was subtracted from individual samples to accomplish normalization. Data presented are representative for n = 4 (for endosomal conditions) or n = 3 (for extracellular and lysosomal conditions); errors are given as standard deviations. Calculations were performed using Origin 7.0 (OriginLab Corporation, Northampton, MA, USA).

### Immunolocalization of cysteine cathepsins in thyroid tissue

Biopsies of human thyroid tissue were taken from the non-disease affected regions after thyroidectomy. The use of human tissue was in compliance with the Helsinki Declaration and was approved to be conducted by WS and KB by the Ethics Commission of the Ärztekammer Bremen (Study 120 as of April 18^th^, 2005) in Germany. Tissue was fixed in 8% paraformaldehyde in 200 mM Hepes buffer, pH 7.4, and mounted in tissue freezing medium after several buffer exchanges. Cryo-sections were taken with a CM 1900 cryostat (Leica, Bensheim, Germany), and immunolabeled following routine procedures [4,7] with sheep anti-human cathepsin B (RD Laboratorien, Diessen, Germany), mouse anti-human cathepsin K (IM 55, Oncogene through Merck Biosciences, Bad Soden, Germany), sheep anti-human cathepsin L (RD Laboratorien), and rabbit anti-human cathepsin S (kindly provided by Dr. E. Weber, Halle, Germany) as primary antibodies, followed by incubation with Alexa 488- or Alexa 546-labeled secondary antibodies (Molecular Probes through Invitrogen, Karlsruhe, Germany). Analysis of mounted tissue was performed by means of confocal laser scanning microscopy (LSM 510 Meta, Zeiss, Oberkochen, Germany).

## Authors' contributions

SJ developed the assay buffer system, performed activity assays, immunoblotting, ELISAs and statistical analyses, analyzed the localization studies, and drafted the manuscript. SJK performed N-terminal sequencing and analyzed cleavage site sequences. NMK participated in the development of the activity assay buffer system. WS contributed to the cathepsin localization study and provided thyroid tissue. DB purified human cathepsins K and S and provided advice in data interpretation. DT and ST discussed experimental setups and contributed in drafting the manuscript. KB devised the study and its design, and drafted the manuscript together with SJ. All authors read and approved the final manuscript.
